# Mucoepidermoid Carcinoma of the Parotid Gland Presenting as a Fungating Exophytic Mass: A Surgical Challenge Rooted in Extensive Nerve Encasement

**DOI:** 10.7759/cureus.10990

**Published:** 2020-10-16

**Authors:** Talal Almas, Salman Hussain, Maryam Ehtesham, Reema Alsufyani, Muhammad Kashif Khan

**Affiliations:** 1 Internal Medicine, Royal College of Surgeons in Ireland, Dublin, IRL; 2 Surgical Oncology, Federal Government Poly Clinic (Post Graduate Medical Institute), Islamabad, PAK; 3 Surgical Oncology, Maroof International Hospital, Islamabad, PAK

**Keywords:** mucoepidermoid, exophytic, marginal mandibular nerve, parotid gland

## Abstract

Parotid glands, which are predominantly composed of serous acini, are the largest salivary glands in the human body. Mucoepidermoid carcinoma (MEC) of the parotid gland is the most common parotid tumour that routinely presents as a painless, fixed mass below the ears. However, its presentation as a fungating mass remains exceedingly rare. Due to the intimate anatomical relationship between the parotid gland and the facial nerve, parotid malignancies can culminate in facial nerve palsy, posing an onerous surgical challenge. In this paper, we chronicle the case of a male who presented with a fungating/exophytic mass and facial nerve weakness. A superficial parotidectomy was performed, and the eventual histopathological workup divulged an advanced mucoepidermoid parotid carcinoma entirely encasing the marginal mandibular nerve. Consequently, the marginal mandibular nerve was sacrificed, alluding to the remarkable surgical challenge encountered. Clinically, this manifested as an impairment of the motor function in the patient's left lower lip.

## Introduction

Salivary gland tumours account for 3%-4% of all head and neck neoplasms. Of all the salivary gland neoplasms, tumours originating from the parotid gland are the most prevalent, comprising 70% of all salivary gland tumours [1]. Notably, the incidence of parotid tumour is between one and three cases per 100,000 every year, with a majority presenting in patients between the ages of 18 and 75 years [2]. Approximately 75% of parotid tumours are benign; the remaining 25% are malignant [1]. Although most tumours of the parotid gland are benign, mucoepidermoid carcinoma (MEC) is noted to be the most common malignant parotid tumour [3]. Current medical literature supports the idea that prior exposure to ionising radiation and familial predisposition to parotid cancer can increase the likelihood of a subsequent parotid malignancy [4]. MEC usually presents as a painless, fixed mass below the ears; however, its presentation as a fungating exophytic mass remains exceedingly rare. We hereby elucidate an interesting case of a patient who presented with an exophytic outgrowth of the parotid gland. A superficial parotidectomy was performed, with the subsequent histopathological workup divulging a diagnosis of high-grade MEC of the parotid gland. Due to the extensive encasement of the facial nerve by the tumour, the marginal mandibular nerve had to be sacrificed, a notion that sensitizes clinicians to the imperativeness of prompt detection and early surgical intervention.

## Case presentation

**Figure 1:**
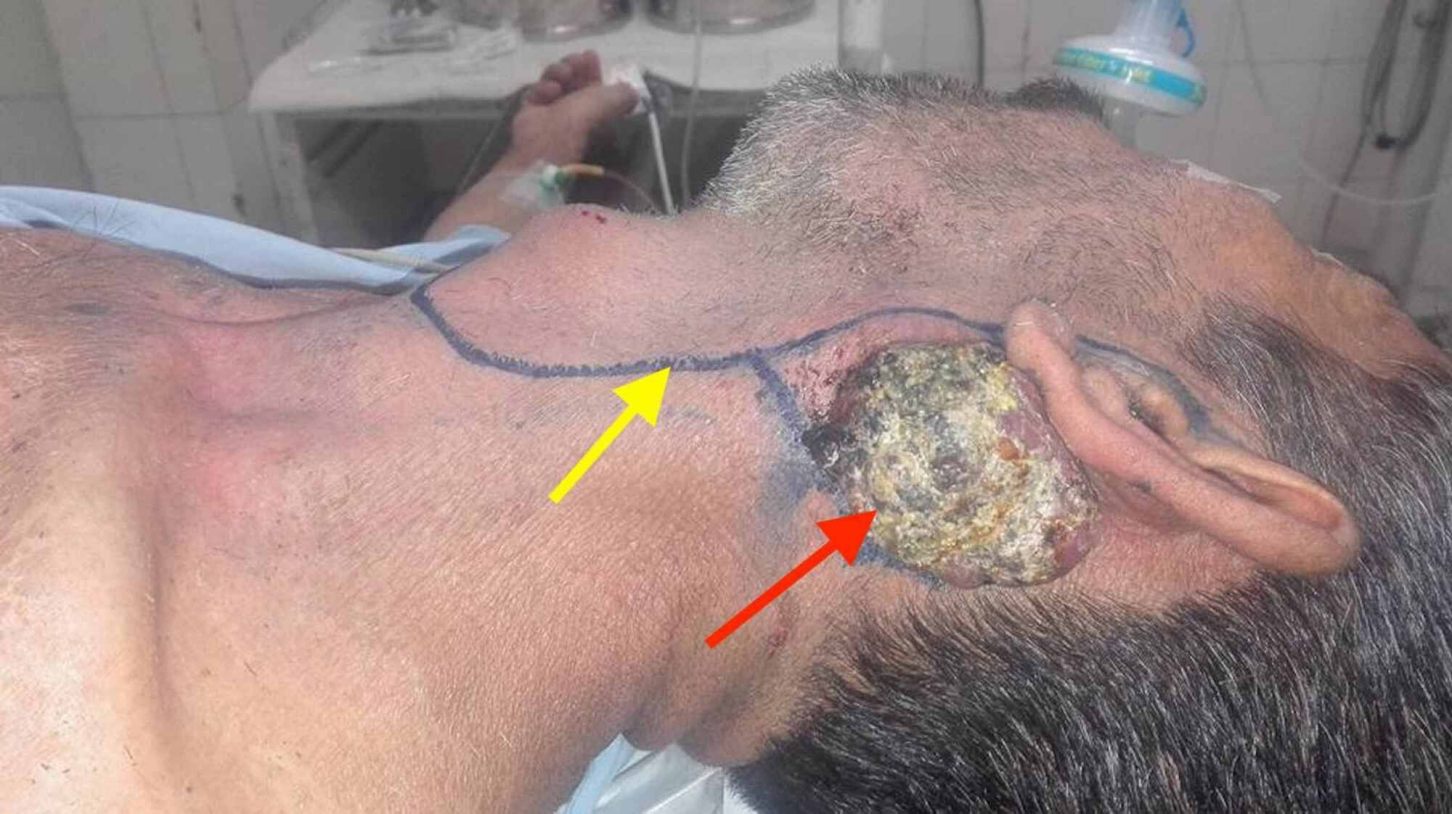
A depiction of the patient's exophytic, fungating parotid mass (red arrow). A Blair incision was thus planned, with the margins delineated preoperatively (yellow arrow).

We elucidate an intriguing case of a 45-year-old male who presented to our medical centre with a six-month history of an increasing swelling below his left ear. Of note, the patient’s prior medical history was unremarkable, with no prior peripheral stigmata of parotid disease. The patient also had no other comorbidities. Notably, the patient’s past surgical history included an uneventful laparoscopic appendectomy performed 10 years ago. Upon presentation, the patient appeared well, with a normal body habitus, and no obvious indication of respiratory distress. A meticulous physical examination of the patient’s neck was thus performed, and identified prominent cervical lymphadenopathy. A thorough medical history was thus obtained and was remarkable. Imperatively, the patient had not had prior exposure to ionising radiation. In order to further evaluate the patient’s cervical lymphadenopathy, a thyroid function panel was ordered and turned out unremarkable. Clinical examination of the facial nerve divulged nerve weakness, alluding to the possibility of an underlying parotid malignancy. Thereafter, a computed tomography (CT) scan was performed and demonstrated an exophytic growth below the left ear with particularly prominent level 2 and level 3 lymphadenopathy (Figure 1). 

**Figure 2:**
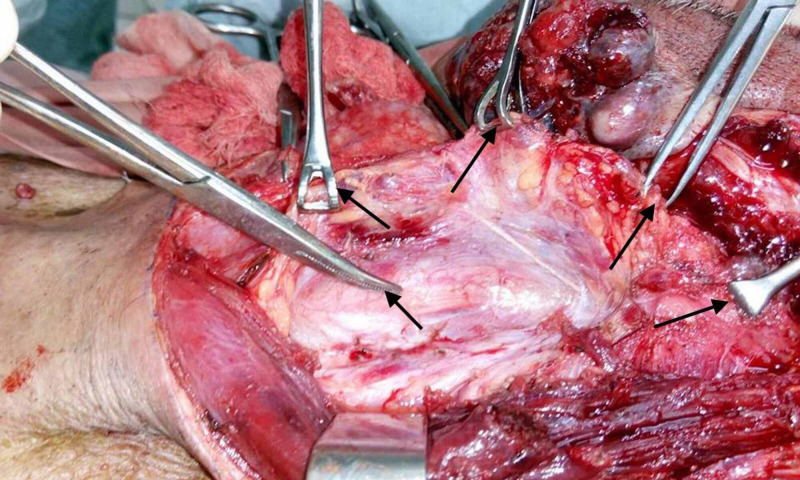
An intraoperative image showing the Babcock holding the specimen artery forceps (black arrows) at the carotid artery after excising the tumour from the underlying cervical nerves.

A fine-needle aspiration (FNA) of the parotid gland revealed diffuse infiltration of the gland by malignant mucinous and squamous cells. Thereafter, a superficial parotidectomy with neck dissection level 1 through to level 4 was planned and performed (Figure 2). 

**Figure 3:**
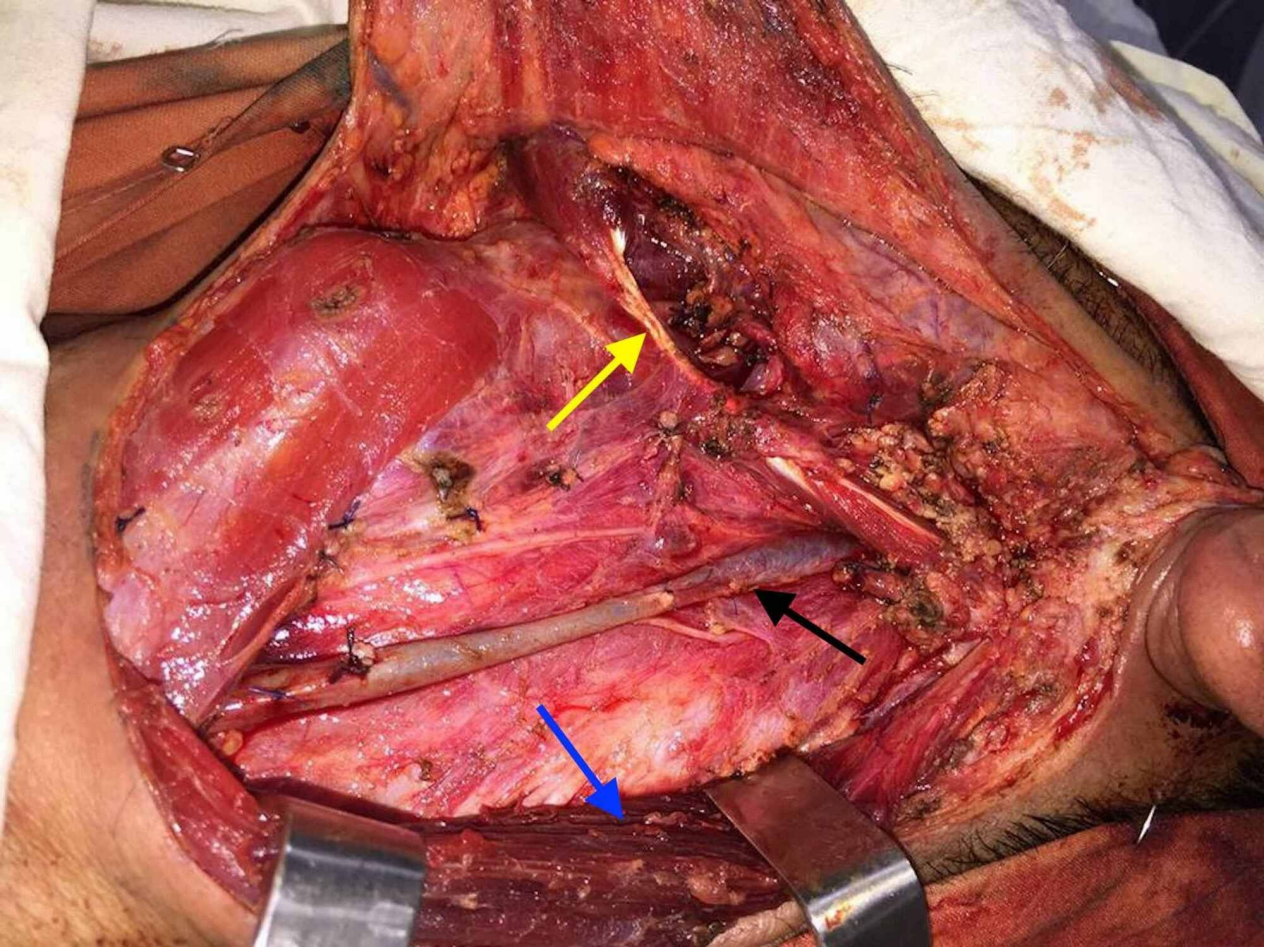
An intraoperative image showing the digastric muscle (yellow arrow), the IJV (black arrow), and the SCM muscle (blue arrow). IJV: internal jugular vein; SCM: sternocleidomastoid

During the procedure, extensive encasement of the facial nerve by the high-grade tumour was noted. The main trunk of the facial nerve was spared; however, the tumour was noted to extensively bore into the lower division of the facial nerve, fomenting a surgical dilemma. After extensive surgical deliberation and due consideration of all available options, a decision was made to resect and in turn, sacrifice the marginal mandibular branch of the facial nerve. The intraoperative image of the neck dissection, along with the underlying anatomical structures, is delineated (Figure 3).

**Figure 4:**
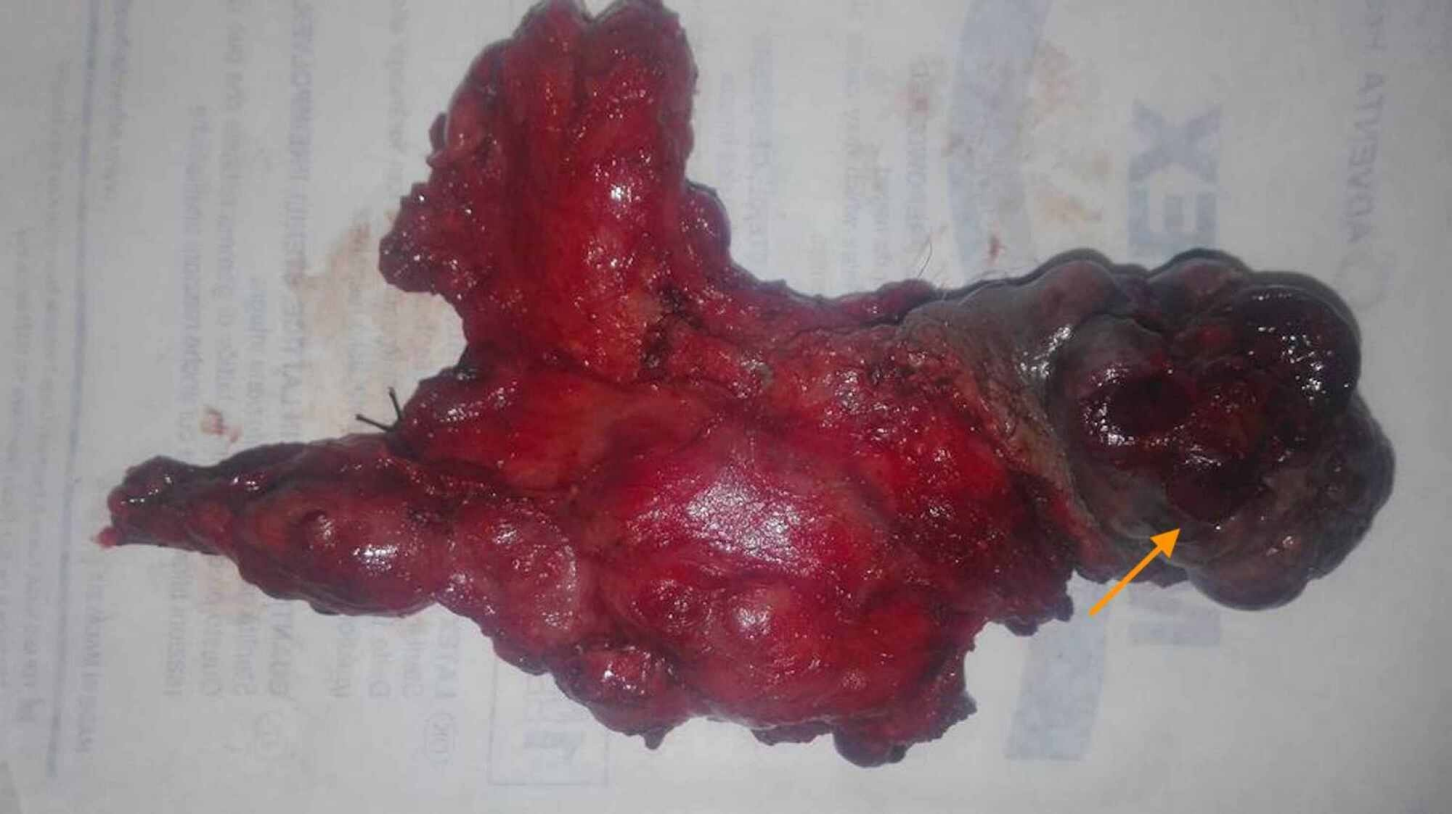
The gross morphology of the resected specimen (orange arrow).

The parotidectomy yielded the specimen shown in Figure 4. 

**Figure 5:**
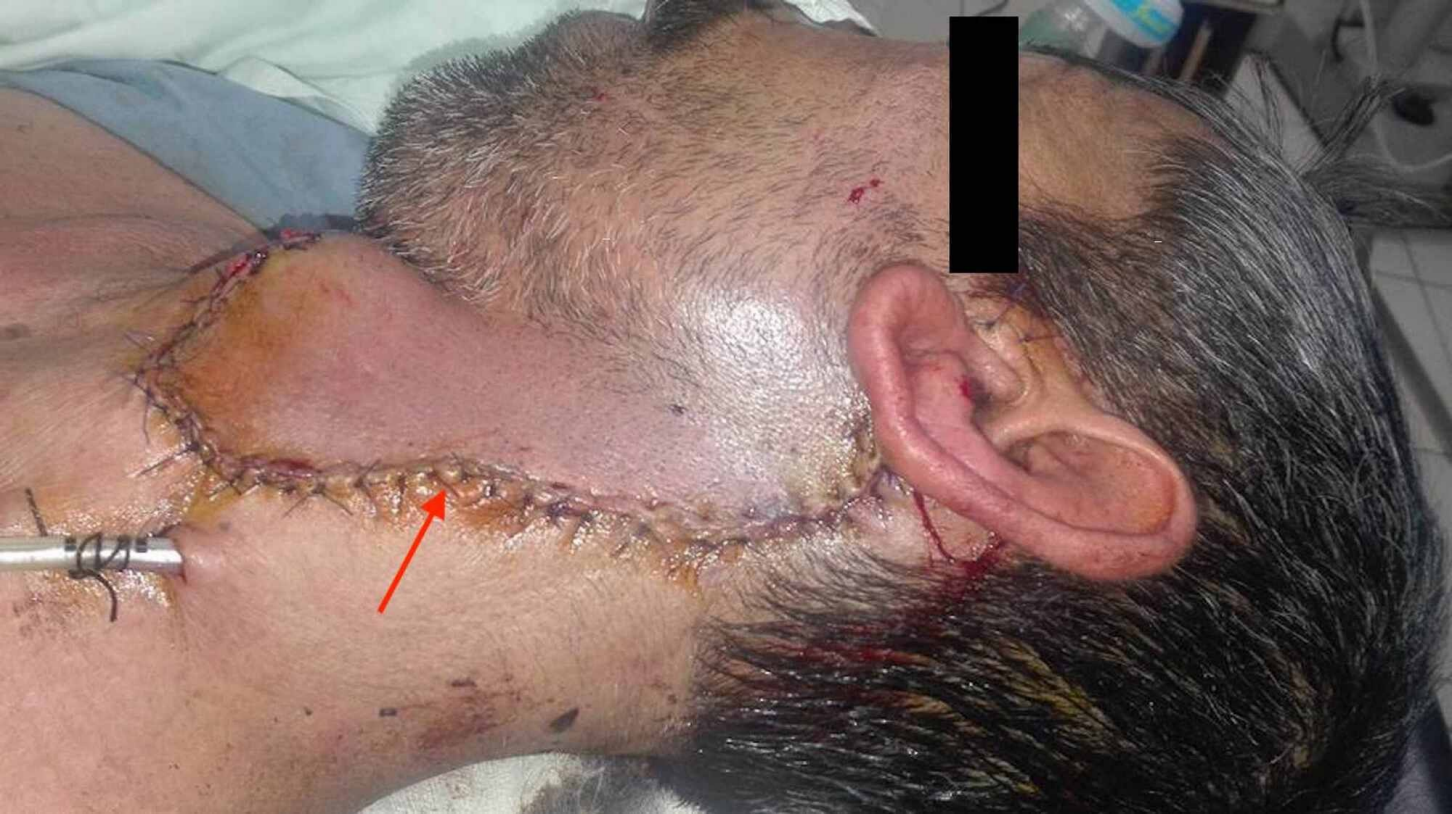
Postoperative image showing the wound closure achieved (red arrow).

Despite the advanced tumour stage, the upper trunk of the facial nerve and the buccal branch were preserved. Pertinently, achieving wound closure posterior to the patient’s left ear was particularly challenging. In order to combat this, flap-rotation was performed and facilitated prompt closure of the wound (Figure 5). 

**Figure 6:**
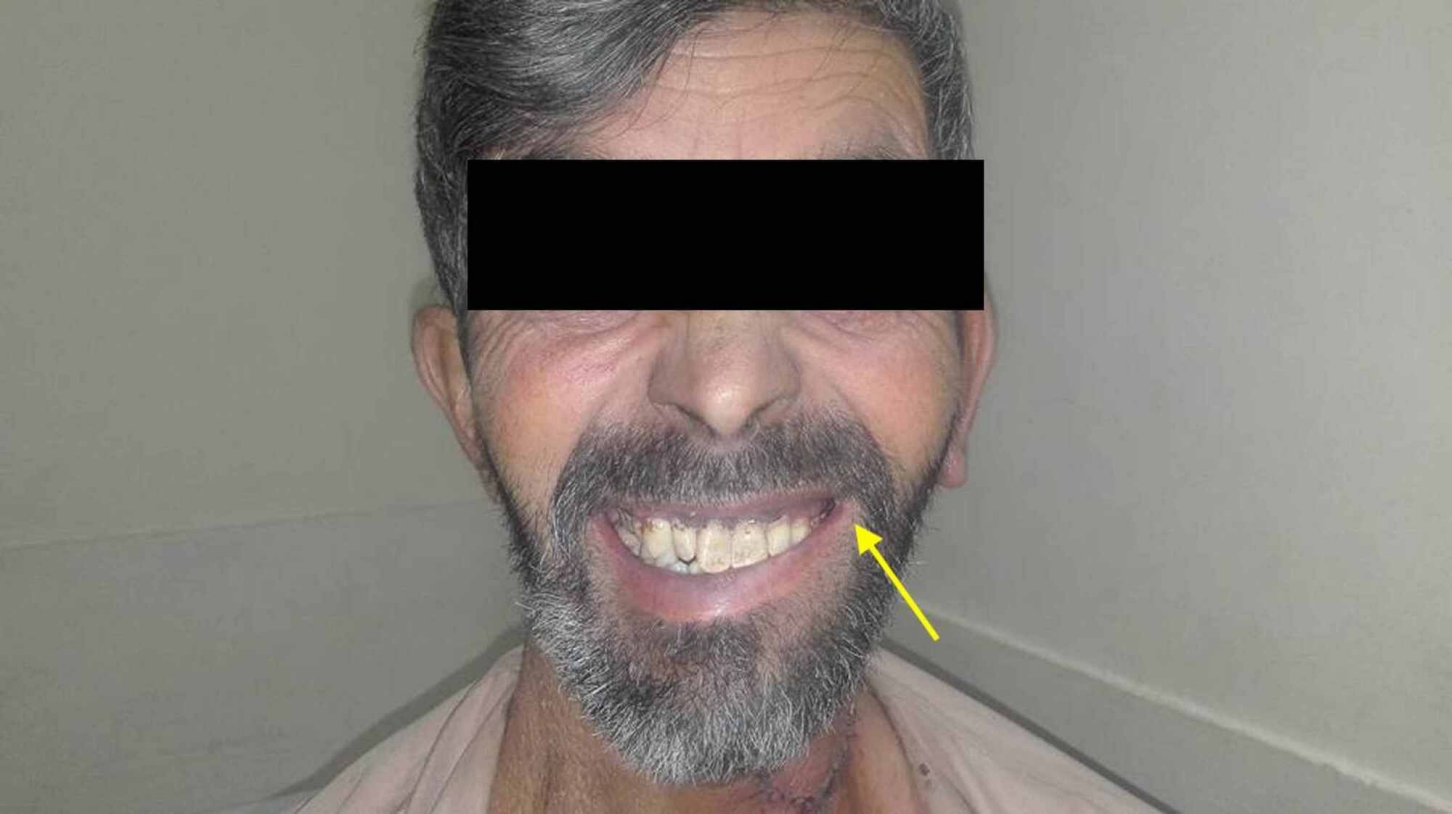
An image obtained at the follow-up visit shows mild motor function impairment of the left lower lip (yellow arrow).

The patient was discharged on the postoperative day five with mild weakness of the lower lip and no other postoperative complications. On a subsequent follow-up visit, weakness of the lower lip was observed (Figure 6). 

## Discussion

Salivary gland malignancies comprise less than 5% of all head and neck malignancies [5]. MEC is the most common malignant salivary gland tumour, usually affecting the minor salivary glands and the parotid gland [6]. MEC accounts for approximately 50% of all parotid malignancies, boasting an incidence rate of 2.3 per 1,000,000 [7,8]. MEC is noted to have a slight predilection to afflicting females, usually affecting adults in their fourth to sixth decades, with the highest prevalence during the fifth decade of life [9]. The origin of MEC is thought to be the pluripotent cells of the excretory ducts of glandular structures [6]. The diagnosis of MEC requires the identification of three cell types, including the epidermoid, mucous, and intermediate cells, each occurring at different proportions, leading to a spectrum of clinical and pathologic behaviours [10].

MEC is classified histopathologically into three grades, which include low, intermediate, and high grades. Low-grade MEC, observed in 48% of the cases, is more common than high grade, which is noted in 38.7% of the cases, while intermediate grade is the least common and is observed in merely 13.3% of the total cases [9]. Histopathologically, they are classified on the basis of the degree of cytological atypia, amount of cyst formation, and the relative number of intermediate, epidermoid and mucous cells [11]. High-grade tumours are considered to be malignant tumours with a poorer prognosis, and usually contain more squamous cells. On the other hand, low-grade tumours are considered less aggressive, and contain a higher proportion of cystic spaces lined with mucous cells [12].

MEC presents clinically as a slow, painlessly enlarging mass mimicking a pleomorphic adenoma, or other benign neoplasms. High-grade malignancies may present as a rapidly growing painless mass infiltrating adjacent structures, thereby culminating in facial nerve palsy. High-grade malignancies are also associated with extra-oral ulceration and metastasis to lymph nodes, lungs, and bone [11]. Nevertheless, the presentation of high-grade MEC as an exophytic, fungating mass remains exceedingly rare. Surgical excision of the tumour, through the means of a superficial parotidectomy, remains the cornerstone of treatment. While low-grade tumours are routinely amenable to local surgical resection, high-grade tumours are usually treated with a wide resection of the tumour [13]. 

The facial nerve traverses the parotid gland and gives rise to five branches herein, illustrating the intimate relation between the facial nerve and the parotid gland. As such, the facial nerve and its branches are particularly susceptible to damage during parotidectomy. It is therefore crucial to correctly identify the extent of invasion using pre-operative imaging in order to preserve the facial nerve. In certain cases where the tumour is noted to invade the facial nerve, damage might be inevitable, eventually leading to paralysis of the facial muscles and loss of tone in the affected muscles [12]. In our case, the main trunk of the facial nerve was not involved and was spared, while the lower division of the facial nerve was invaded by the tumour; the marginal mandibular and cervical branches of the facial nerve were thus sacrificed. Clinically, this presented as an impairment of the patient’s lower lip motor function. Early detection of malignant parotid tumours through the means of multimodal imaging, including magnetic resonance imaging and computed tomography, is therefore pivotal in yielding favourable disease outcomes. 

## Conclusions

While the vast majority of parotid gland tumours are benign, malignant tumours include neoplasms such as MEC. An enlarging, high-grade MEC presents clinically as a painless, fixed mass; however, its presentation as a fungating, exophytic mass remains exceedingly rare. Left undetected, MECs can proliferate to encase the facial nerve, necessitating the resection of the nerve or one of its branches. Early detection of such tumours through the means of multimodal imaging, along with a multidisciplinary team approach and meticulous clinical history, is therefore imperative in portending favourable disease outcomes.
